# Robust Kalman filter for heavy-tailed process and measurement noises

**DOI:** 10.1038/s41598-026-50388-3

**Published:** 2026-05-07

**Authors:** Qianxin Wang, Chenwang Ye, Guobin Chang, Conghao Tang, Xun Zhang

**Affiliations:** 1 School of Environment Sciences and Spatial Informatics, China University of Mining and Technology, Xuzhou, 221116 China; 2 State Key Laboratory of Spatial Datum, Xi’an, 710054 China

**Keywords:** Kalman filter, Robust, Heavy tail, M-estimator, Engineering, Mathematics and computing

## Abstract

Measurements and dynamic process models are often contaminated by errors or noises. In many real applications, for simplicity of computations, it is assumed such errors are random and follow a normal distribution. However, the normal-distribution assumption can only be an approximation; and the true distribution can be a heavier tailed one rather than the assumed normal one. Some optimal estimators, including Kalman filters, can face serious performance degradation in the presence of heavy-tailed distributions. Previous robust Kalman filtering schemes are not directly targeted on these two types of noises, especially the process noise; instead, the predicted state estimation error is considered together with the measurement noise. The topic of the current study is the robust modification of the Kalman filtering against heavy-tailed distributions of both the process and measurement noises. A new robust Kalman filter is proposed. The new filter is simple, in the sense that it has the same structure of a standard Kalman filter. It is highly automated, in the sense that it can be initialized by the standard Kalman filtering. It is effective as it is insensitive to heavy-tailed distributions of both the process and measurement noises. The proposed method is general and flexible, in the sense that it can be readily modified to consider reliable prior distributional information. Simulation of a tracking application and real data analysis of an integrated GNSS and INS application demonstrate the effectiveness of the proposed method. The tracking simulation shows the proposed method reduces the positioning Root Mean Squared Error (RMSE) by 11.1% compared with the standard Kalman filter, and the reduction can reach 20.0% with five iterations. The real GNSS/INS navigation experiment achieves up to 22% reduction in positioning RMSE and obvious improvements in velocimetry performance relative to the standard Kalman filter.

## Introduction

The standard Kalman filter is a classical optimal estimation method for linear discrete-time state space systems and serves as the theoretical foundation for numerous state estimation problems in engineering applications^1^. It is constructed on linear dynamic process and measurement models, with core assumptions that process and measurement noises are mutually independent zero-mean white noise sequences with known covariance matrices. Based on a given initial state estimate and its corresponding covariance matrix, the Kalman filter obtains the optimal state estimate at each epoch through a recursive calculation framework composed of two core stages: state prediction and state update. The prediction stage derives the a priori state estimate and its covariance matrix using the state transition relationship, while the update stage corrects the a priori estimate with real-time measurement information to generate the a posteriori state estimate^2,3^.

For linear regression models, the weighted least-squares estimator is proven to be the Best Linear Unbiased Estimator by the Gauss-Markov theorem^4^. Similarly, the Kalman filter yields the minimum-variance Best Linear Unbiased Estimator for linear state space systems, and this optimality holds without any constraints on noise distribution^5^. When process and measurement noises follow a normal distribution, the Kalman filter is further optimal in the sense of maximum likelihood and Bayesian estimation, which is the key reason for the widespread adoption of the normal distribution assumption in Kalman filter design^6^.

Inevitable modeling uncertainties mean most mathematical models are only simplified abstractions of practical engineering realities, and this applies equally to the state space model of the Kalman filter^7–9^. The normal distribution assumption of process and measurement noises is a prerequisite for the Kalman filter to achieve maximum likelihood or Bayesian optimality, yet this assumption is only an approximation in actual applications. In practice, the real noise distribution is highly likely to deviate from the normal distribution, and such deviations can be categorized into light-tailed and heavy-tailed non-normal distributions. This study focuses exclusively on heavy-tailed noise distributions because the light-tailed distribution has almost no adverse impact on the estimation performance of the standard Kalman filter, while the heavy-tailed distribution can lead to severe and unacceptable performance degradation of the filter^10^.

To address the performance degradation of the standard Kalman filter under non-Gaussian heavy-tailed noises and complex measurement uncertainties, relevant robust schemes have been widely studied. Non-stationary heavy-tailed measurement noise and unknown time-varying measurement loss probability in linear systems are simultaneously handled via Gaussian-Student’s t-mixture distribution modeling and variational Bayesian-based joint state-parameter estimation^11^. A Multivariate Laplace distribution-based strategy is further developed to avoid tedious degree-of-freedom parameter tuning, realizing adaptive covariance inference for both heavy-tailed process and measurement noises^12^. For nonlinear systems, a robust Gaussian approximate filter and smoother with Normal-Gamma-Beta distribution is constructed to achieve synchronous unknown latency probability identification and heavy-tailed noise suppression via variational Bayesian inference^13^. A novel multivariate Laplace distribution-based Gaussian approximate filter is proposed to tackle the colored heavy-tailed measurement noise problem in nonlinear systems, which converts colored noise into white heavy-tailed noise via measurement differencing and state extension and performs joint inference of state and noise covariance using variational Bayesian methodology^14^. These studies have laid a theoretical foundation for non-Gaussian robust filtering, while a research gap remains in targeted separate processing of heavy-tailed process and measurement noises for further performance improvement.

When robust estimators severely down-weight or isolate extreme heavy-tailed outliers, the effective observation sequence essentially degenerates into a sparse or missing data scenario. To tackle such inherent challenges, advanced data-driven frameworks have been proposed, including continuous-time Hammerstein modeling with missing data using the improved Archimedes optimization algorithm^15^, and system identification with sparse measurement data utilizing the improved marine predators algorithm^16^. Similarly, in macroscopic applications, physics-informed traffic state estimation models have been successfully developed for freeways under sparse observation data^17^. For nonlinear systems with missing data, Bayesian hypothesis testing has been integrated with distributed estimation to achieve robust parameter identification by adaptively distinguishing valid measurements from missing ones^18^.

When the true noise distribution is heavy-tailed and deviates from the assumed normal distribution, the estimation accuracy of the standard Kalman filter decreases significantly, which is defined as the robustness deficiency problem in statistical estimation^19^. The core objective of designing a robust estimator is to achieve distributional robustness, which means maintaining stable and reliable estimation performance when the actual noise distribution deviates from the pre-assumed theoretical model^20^.

The deviation between the normal distribution assumption and the actual noise distribution can be divided into large and small deviations, and this study focuses on small deviations for their higher practical engineering value. Mature high breakdown-point methods have been developed for large deviation cases^21,22^. Small deviations from the normal distribution can be effectively characterized by the general contaminated normal model, whose worst-case scenario is the so-called least favorable distribution. The maximum likelihood estimator under this least favorable distribution is the M-estimator, currently the most widely used robust estimation method^23^. The M-estimator is asymptotically unbiased and has the smallest asymptotic variance among all asymptotically unbiased estimators under a given distribution, and its excellent analytical properties make it the preferred framework for robust filter design^24^. The minimax optimality of the M-estimator, which means achieving the best estimation performance under the worst-case distribution, endows it with the desired robustness against non-normal noise distributions. For more information on robust statistics in general and on M-estimator in specific, one can go to^20^.

To apply the M-estimator principle to Kalman filtering problems, the core step is to replace the non-robust quadratic loss function underlying the standard Kalman filter with a robust loss function^25,26^. From a likelihood perspective, this replacement is equivalent to replacing the non-robust normal distribution assumption with a robust distribution assumption that adapts to noise deviation. The Huber loss function is the most typical robust loss function for this purpose. It modifies the quadratic loss function by retaining the quadratic form for small errors and adopting a linear form for large tail errors, thus realizing robust processing of heavy-tailed noise^23^. Although this study adopts the Huber loss function, many other robust loss functions can be used in practical applications, even though most lack the strict theoretical properties of the Huber loss function^27,28^.

Existing M-estimator-based robust Kalman filter methods typically transform the state update process of the Kalman filter into a least-squares estimation problem of an augmented measurement model, and further introduce a decoupled measurement model through Cholesky decomposition to facilitate robust modification. The robust state estimate is then obtained by minimizing a comprehensive robust loss function for the decoupled model, and the iteratively reweighted least-squares (IRLS) algorithm is the most commonly used numerical method for solving this minimization problem^29^. When using the IRLS algorithm, each iteration can be calculated using the same steps as the standard Kalman filter update, but the a priori covariance matrix and/or measurement noise covariance matrix need to be inflated. However, inflating the a priori covariance matrix essentially implements a fading memory operation, which is often classified as an adaptive or strong-tracking method rather than a pure robust method in existing studies^30–32^.

A notable characteristic of existing robust Kalman filter schemes is that they do not directly target the heavy-tailed characteristics of process and measurement noises individually. Instead, they tend to incorporate the predicted state estimation error into the robust processing framework together with the measurement noise, without explicitly distinguishing the independent sources of these two error components. In fact, the state prediction error can be decomposed into two components: the estimation error of the filtering estimate at the previous epoch and the error caused by the current epoch’s process noise. If the previous epoch’s filtering estimate is obtained via an appropriate robust method, it is reasonable to assume the predicted state vector follows a normal distribution, which means only the process noise component in the prediction error requires robust processing for heavy-tailed characteristics. This indicates that the existing proxy-based robust processing scheme lacks targeting, and a more reasonable approach is to directly treat the heavy-tailed process and measurement noises separately.

To address the above limitations, this study proposes a new robust Kalman filter method that directly targets the heavy-tailed distributions of both process and measurement noises. Based on the M-estimation framework, the proposed method realizes targeted separate processing of heavy-tailed process and measurement noises via a variance-component inflation strategy, retaining the same recursive structure as the standard Kalman filter to ensure easy implementation and low computational complexity. It avoids the complex parameter tuning required by most existing heavy-tailed robust filters, and real integrated Global Navigation Satellite System (GNSS) and Inertial Navigation System (INS) navigation data tests.

The proposed method is presented in Sect. 2, with the derivations detailed in **Appendix A**. In Sect. 3, first, a simple tracking problem simulation is employed to demonstrate the effectiveness of the proposed method; then, the performance of the proposed method is checked in a real GNSS/INS integration. The paper is briefly concluded in Sect. 4.

## A new robust Kalman filter

### Theoretical foundation

First, the basic models and core functions of the standard Kalman filter and M-estimator are given as the theoretical foundation for the proposed method, including the dynamic process model, measurement model, recursive algorithm and Huber loss function. The relevant definitions and mathematical expressions are as follows,

1 $$\left\{ \begin{gathered} {\boldsymbol{x}_k}={\boldsymbol{F}_{k - 1}}{\boldsymbol{x}_{k - 1}}+{\boldsymbol{w}_{k - 1}} \hfill \\ {\boldsymbol{y}_k}={\boldsymbol{H}_k}{\boldsymbol{x}_k}+{\boldsymbol{\eta}_k} \hfill \\ \end{gathered} \right.$$

In (1), integer *k* in the subscripts denotes the epoch index; ***x***_*k*_ denotes the n-dimensional state vector to be estimated in real time in a filtering problem; ***y***_*k*_ denotes the m-dimensional measurement vector which becomes available since the kth epoch; the state transition matrix ***F***_*k*−1_ and the measurement matrix ***H***_*k*_ are known; the process noise ***w***_*k*−1_ and the measurement noise ***η***_*k*_ are assumed to be zero-mean white series, independent of each other; their covariance matrices, denoted as ***W***_*k*−1_ and ***R***_*k*_ are also assumed known.

Let $\:{\widehat{\boldsymbol{x}}}_{i\left|j\right.}$ be an estimate of ***x***_*i*_ with the measurements available up to the *j*th epoch; let ***P***_*i|j*_ be the nominal covariance matrix of the estimate $\:{\widehat{\boldsymbol{x}}}_{i\left|j\right.}$. Given the initial estimate $\:{\widehat{\boldsymbol{x}}}_{0\left|0\right.}$ with its nominal covariance matrix ***P***_0|0_, the following recursive algorithm provides optimal filtering estimates at any epoch *k* > 0,

2 $$\left\{ \begin{gathered} {{\boldsymbol{\hat {x}}}_{k\left| {k - 1} \right.}}={\boldsymbol{F}_{k - 1}}{{\boldsymbol{\hat {x}}}_{k - 1\left| {k - 1} \right.}} \hfill \\ {\boldsymbol{P}_{k\left| {k - 1} \right.}}={\boldsymbol{F}_{k - 1}}{\boldsymbol{P}_{k - 1\left| {k - 1} \right.}}\boldsymbol{F}_{{k - 1}}^{{\mathrm{T}}}+{\boldsymbol{W}_{k - 1}} \hfill \\ {\boldsymbol{K}_k}={\boldsymbol{P}_{k\left| {k - 1} \right.}}\boldsymbol{H}_{k}^{{\mathrm{T}}}{\left( {{\boldsymbol{H}_k}{\boldsymbol{P}_{k - 1\left| {k - 1} \right.}}\boldsymbol{H}_{k}^{{\mathrm{T}}}+{{\boldsymbol{R}}_k}} \right)^{ - 1}} \hfill \\ {{\boldsymbol{\hat {x}}}_{k\left| k \right.}}={{\boldsymbol{\hat {x}}}_{k\left| {k - 1} \right.}}+{\boldsymbol{K}_k}\left( {{\boldsymbol{y}_k} - {\boldsymbol{H}_k}{{\boldsymbol{\hat {x}}}_{k\left| {k - 1} \right.}}} \right) \hfill \\ {\boldsymbol{P}_{k\left| k \right.}}={\boldsymbol{P}_{k\left| {k - 1} \right.}} - {\boldsymbol{K}_k}{\boldsymbol{H}_k}{\boldsymbol{P}_{k\left| {k - 1} \right.}} \hfill \\ \end{gathered} \right.$$

where ***K***_*k*_ denotes the Kalman gain matrix.

The above algorithm is the standard Kalman filter^1^.

The Huber loss function is the core robust loss function adopted in this study, which is the negative log-likelihood function of the least favorable distribution in the contaminated normal model. Its mathematical definition is^23^,

3 $$\rho \left( e \right)=\left\{ \begin{gathered} \frac{1}{2}{e^2}{\text{, }}\left| e \right| \leqslant c \hfill \\ c\left| e \right| - \frac{1}{2}{c^2}{\mathrm{,}}\left| e \right|>c \hfill \\ \end{gathered} \right.$$

where ***e*** represents the standardized error. The threshold parameter *c* is set to 1.345 according to the classical asymptotic relative efficiency theory in robust statistics. Specifically, this value ensures that the estimator maintains 95% efficiency of the standard Kalman filter under Gaussian noise while achieving strong robustness against heavy-tailed noise. Regarding parameter sensitivity, the algorithm maintains highly stable performance when *c* varies within the typical neighborhood [1.0, 1.5]. A smaller *c* more aggressively downweights observations, improving robustness at the cost of nominal efficiency, whereas a larger *c* behaves conversely. Thus,1.345 is adopted as the optimal theoretical trade-off for small-deviation models. Intuitively from (3), the Huber loss function is a modified version of the non-robust quadratic loss function; the modification is made at two tails, with the center unchanged. As a note here, even though the Huber loss (3) is adopted in this contribution, many other loss functions can also be adopted from a practical viewpoints^27,28^.

In the formulation of some M-estimator based robust Kalman filters, as the first step, the update of the Kalman filter is viewed as the least-squares estimation for the following augmented measurement or regression model,

4 $$\left[ {\begin{array}{*{20}{c}} {{{\boldsymbol{\hat {x}}}_{k\left| {k - 1} \right.}}} \\ {{\boldsymbol{y}_k}} \end{array}} \right]=\left[ {\begin{array}{*{20}{c}} {{\mathbf{I}_n}} \\ {{\boldsymbol{H}_k}} \end{array}} \right]{\boldsymbol{x}_k}+\left[ {\begin{array}{*{20}{c}} {{\boldsymbol{\boldsymbol{\upvarepsilon}}_k}} \\ {{\boldsymbol{\eta}_k}} \end{array}} \right]{\text{, with }}\operatorname{cov} \left( {\left[ {\begin{array}{*{20}{c}} {{\boldsymbol{\boldsymbol{\upvarepsilon}}_k}} \\ {{\boldsymbol{\eta}_k}} \end{array}} \right]} \right)=\left[ {\begin{array}{*{20}{c}} {{\boldsymbol{P}_{k\left| {k - 1} \right.}}}&0 \\ 0&{{{\boldsymbol{R}}_k}} \end{array}} \right]$$

where **I**_n_ denotes the n⋅n identity matrix, and ε_k_ denotes the state prediction error.

The equivalence between the least-squares estimation for (4) and the update of the Kalman filtering can be proved via the matrix inversion lemma, also called the Sherman–Morrison–Woodbury formula^3^; the proof is omitted here for brevity.

Let ***P***_*k|k−*1_=***AA***^T^ and ***V***_*k*_=***SS***^T^, denoting the Cholesky decompositions. Introduce the following decoupled measurement model,

5 $$\boldsymbol{l}=\left[ {\begin{array}{*{20}{c}} {{{\boldsymbol{A}}^{ - 1}}{{\boldsymbol{\hat {x}}}_{k\left| {k - 1} \right.}}} \\ {{\boldsymbol{S}^{ - 1}}{\boldsymbol{y}_k}} \end{array}} \right]=\left[ {\begin{array}{*{20}{c}} {{{\boldsymbol{A}}^{ - 1}}} \\ {{\boldsymbol{S}^{ - 1}}{\boldsymbol{H}_k}} \end{array}} \right]{\boldsymbol{x}_k}+\left[ {\begin{array}{*{20}{c}} {{{\boldsymbol{A}}^{ - 1}}{\boldsymbol{\boldsymbol{\upvarepsilon}}_k}} \\ {{\boldsymbol{S}^{ - 1}}{\boldsymbol{\eta}_k}} \end{array}} \right]=\boldsymbol{G}{\boldsymbol{x}_k}+\boldsymbol{\boldsymbol{\upxi}}{\text{, with }}\operatorname{cov} \left( \boldsymbol{\boldsymbol{\upxi}} \right)={\mathbf{I}_{m+n}}$$

where ***l G***, and $\boldsymbol{\boldsymbol{\upxi}}$ are the normalized augmented measurement vector, normalized augmented measurement matrix, and normalized augmented error vector, respectively, obtained via Cholesky decomposition.

The least-squares estimate for (5) coincides with that for (4) and hence with the Kalman filtering estimate. In the M-estimation, the estimate can be defined as the minimizer of the following loss function,

6 $$\varphi \left( {{\boldsymbol{x}_k}} \right)=\sum\limits_{{i=1}}^{n} {{\rho _1}\left( {{l_i} - \boldsymbol{g}_{i}^{{\mathrm{T}}}{\boldsymbol{x}_k}} \right)} +\sum\limits_{{i=n+1}}^{{m+n}} {{\rho _2}\left( {{l_i} - \boldsymbol{g}_{i}^{{\mathrm{T}}}{\boldsymbol{x}_k}} \right)}$$

where *l*_*i*_ and ***g***_*i*_^T^ are the *i*th row of ***l*** and ***G***, respectively. When both *ρ*_1_ and *ρ*_2_ are quadratic, the minimizer of (6) is the least-squares estimate, and hence the Kalman filtering estimate. One can choose other functions for *ρ*_1_ and/or *ρ*_2_, to achieve robustness against non-normality of the predicted state estimate and/or the measurement^33–35^. Numerical minimization of this loss function usually requires iterations; with the IRLS algorithm, each iteration follows the standard Kalman filter update steps but with inflated ***P***_*k|k*−1_ and/or ***V***_*k*_^29^.

The state prediction error ***ε***_*k*_ in (4) is the sum of two terms, shown as follows,

7 $${\boldsymbol{\boldsymbol{\upvarepsilon}}_k}={\boldsymbol{F}_{k - 1}}{\mathrm{d}}{\boldsymbol{\hat {x}}_{k - 1\left| {k - 1} \right.}}+{\boldsymbol{w}_{k - 1}}$$

where ${\mathrm{d}}{\boldsymbol{\hat {x}}_{k - 1\left| {k - 1} \right.}}$ denotes the estimation error in the filtering estimate ${\boldsymbol{\hat {x}}_{k - 1\left| {k - 1} \right.}}$. If ${\boldsymbol{\hat {x}}_{k - 1\left| {k - 1} \right.}}$ is obtained with an appropriate robust method at the previous epoch, it is more appropriate to assume that the predicted state vector has a normal distribution, rather than a heavy-tailed non-normal one. Or in other words, it is only the second term in the right side of (7) that needs to be considered in a robust way. Therefore, a new method is proposed in this contribution to directly treat the heavy tailed process and/or the measurement noises, without resorting to the prediction error as a proxy in (7).

### The proposed method

On the basis of the above basic models and functions, the proposed robust Kalman filter method is defined as the solution to a minimization problem. Let ***W***_*k*−1_=***QQ***^T^, denoting the Cholesky decomposition of the process noise covariance matrix; under the assumptions made to the state space mode (1), the matrix ***Q*** is known. As in the previous section, let ***R***_*k*_=***SS***^T^, with known ***R***_*k*_. Let $$\left\{ \begin{gathered} \boldsymbol{r}={\boldsymbol{Q}^{ - 1}}{\boldsymbol{w}_{k - 1}} \hfill \\ \boldsymbol{s}={\boldsymbol{S}^{ - 1}}{\boldsymbol{\eta}_k} \hfill \\ \end{gathered} \right.$$; they are normalized noise vectors, which are unknown, but with known covariance matrices $$\left\{ \begin{gathered} {\mathrm{cov}}\left[ \boldsymbol{r} \right]={\mathbf{I}_n} \hfill \\ {\mathrm{cov}}\left[ \boldsymbol{s} \right]={\mathbf{I}_m} \hfill \\ \end{gathered} \right.$$.

Before proceeding, it is necessary to clarify the context of the known and unknown quantities. In practical GNSS/INS applications, the exact numerical realizations of noise vectors at any specific epoch are inherently unknown. However, Q and ***R***_*k*_ are treated as known inputs. This assumption is highly realistic, as these matrices are dynamically determined through established stochastic modeling. For instance, GNSS measurement covariance is typically modeled in real-time based on satellite elevation angles, while INS process noise covariance is derived from pre-calibrated IMU parameters. Under the nominal linear Gaussian modeling assumption, these normalized noise vectors *r* and *s* are assumed to strictly follow a standard multivariate normal distribution, i.e., ${\rm N}\left( {0,\mathbf{I}} \right)$, where **I** denotes the identity matrix. It is the possible deviation of the true distributions from this nominal standard normal baseline (i.e., the presence of heavy-tailed non-normality) that motivates the M-estimation formulation in the following steps. Let $$\left\{ \begin{gathered} {\boldsymbol{l}_1}={\boldsymbol{Q}^{ - 1}}{{\boldsymbol{\hat {x}}}_{k\left| {k - 1} \right.}} \hfill \\ {\boldsymbol{l}_2}={\boldsymbol{S}^{ - 1}}{\boldsymbol{y}_k} \hfill \\ \end{gathered} \right.$$, denoting the normalized measurement vectors which are known. Let $$\left\{ \begin{gathered} {\boldsymbol{L}_1}={\boldsymbol{Q}^{ - 1}} \hfill \\ {\boldsymbol{L}_2}={\boldsymbol{S}^{ - 1}}{\boldsymbol{H}_k} \hfill \\ \end{gathered} \right.$$, denoting the corresponding measurement matrices, which are known. Let ${\boldsymbol{n}}={\boldsymbol{Q}^{ - 1}}{\boldsymbol{F}_{k - 1}}{\mathrm{d}}{\boldsymbol{\hat {x}}_{k - 1\left| {k - 1} \right.}}$ denoting the part of errors in ***l***_1_ caused by the errors of the filtering estimate at the previous epoch, which is unknown. Readily, the covariance matrix of this error vector is $\boldsymbol{U}=\operatorname{cov} \left[ {\boldsymbol{n}} \right]={\boldsymbol{Q}^{ - 1}}{\boldsymbol{F}_{k - 1}}{\boldsymbol{P}_{k - 1\left| {k - 1} \right.}}\boldsymbol{F}_{{k - 1}}^{{\mathrm{T}}}{\boldsymbol{Q}^{ - {\mathrm{T}}}}$, which is known. One can have the following measurement model,

8 $$\left\{ \begin{gathered} {\boldsymbol{l}_1}={\boldsymbol{L}_1}{\boldsymbol{x}_k}+{\boldsymbol{n}}+\boldsymbol{r} \hfill \\ {\boldsymbol{l}_2}={\boldsymbol{L}_2}{\boldsymbol{x}_k}+\boldsymbol{s} \hfill \\ \end{gathered} \right.$$

The least-squares estimation for the model (8) is exactly the same as that for the model (4) or (5), and hence to the standard Kalman filtering; these equivalences can be proved via straightforward matrix-vector algebras and hence omitted here. According to the basic M-estimator theory, a generalized filtering estimate of ***x***_*k*_ is defined as the minimizer of the following loss function,

9 $$\phi ={\rho _1}\left( \boldsymbol{r} \right)+{\rho _2}\left( \boldsymbol{s} \right)+\frac{1}{2}{{\boldsymbol{n}}^{\mathrm{T}}}{\boldsymbol{U}^{ - 1}}{\boldsymbol{n}}+\boldsymbol{\boldsymbol{\uplambda}}_{1}^{{\mathrm{T}}}\left( {{\boldsymbol{l}_1} - {\boldsymbol{L}_1}{\boldsymbol{x}_k} - {\boldsymbol{n}} - \boldsymbol{r}} \right)+\boldsymbol{\boldsymbol{\uplambda}}_{2}^{{\mathrm{T}}}\left( {{\boldsymbol{l}_2} - {\boldsymbol{L}_2}{\boldsymbol{x}_k} - \boldsymbol{s}} \right)$$

where ***λ***_1_ and ***λ***_2_ are the unknown Lagrange multiplier vectors. The two cost functions involved in (9) are defined element-wise, as follows,

10 $$\left\{ \begin{gathered} {\rho _1}\left( \boldsymbol{r} \right)=\sum\limits_{{i=1}}^{n} {\rho \left( {{r_i}} \right)} \hfill \\ {\rho _2}\left( \boldsymbol{s} \right)=\sum\limits_{{i=1}}^{m} {\rho \left( {{s_i}} \right)} \hfill \\ \end{gathered} \right.$$

If robust estimation is desired, the loss function *ρ*can be chosen as the Huber loss function defined in (3). The loss function as shown in (9) is the departure from the previous M-estimation based robust filter algorithms, see e.g^33–35^, in which the loss function in (6) was utilized, instead.

The derivation of the new solution to the problem of minimizing (9) is detailed in **Appendix A**. From the solution a quite simple iterative algorithm can be proposed. In each iteration, the algorithm is in the same form as a standard Kalman filter in (2) but with modified covariance matrices of the measurement and the process noises.

Let the estimate of the standard Kalman filter in (2) be denoted as ${\boldsymbol{\bar {x}}_k} \leftarrow {\boldsymbol{\hat {x}}_{k\left| k \right.}}$; this will be used to initialize the proposed iterative method. According to **Appendix A**, with the estimated state vector in the immediately previous iteration, denoted as ${\boldsymbol{\bar {x}}_k}$, the estimates of the normalized noises are as follows,

11 $$\left\{ \begin{gathered} \boldsymbol{\bar {r}}={\boldsymbol{Q}^{\mathrm{T}}}{\left( {{\boldsymbol{F}_{k - 1}}{\boldsymbol{P}_{k - 1\left| {k - 1} \right.}}\boldsymbol{F}_{{k - 1}}^{{\mathrm{T}}}+{\boldsymbol{W}_{k - 1}}} \right)^{ - 1}}\left( {{{\boldsymbol{\hat {x}}}_{k\left| {k - 1} \right.}} - {{\boldsymbol{\bar {x}}}_k}} \right) \hfill \\ \boldsymbol{\bar {s}}={\boldsymbol{S}^{ - 1}}\left( {{\boldsymbol{y}_k} - {\boldsymbol{H}_k}{{\boldsymbol{\bar {x}}}_k}} \right) \hfill \\ \end{gathered} \right.$$

In order to simplify the presentation of the proposed algorithm, introduce the following function,

12 $$\omega \left( e \right)=\frac{e}{{\rho ^{\prime}\left( e \right)}}=\left\{ \begin{gathered} 1{\text{, }}\left| e \right| \leqslant c \hfill \\ \frac{{\left| e \right|}}{c}{\text{, }}\left| e \right|>c \hfill \\ \end{gathered} \right.$$

which is called a variance-component inflation function. It is called “inflation” because the function value of *ω*(*e*) is always larger than or equal to 1. It is noted that the above variance-component inflation function is the inverse of the so-called equivalent weight function^36,37^. To be a little more specific, the value of this equivalent weight function is always smaller than or equal to 1. With such weighting, measurements with abnormally large errors will be underweighted in the estimation. It is this underweighting strategy that brings the desired robustness.

Introduce the following covariance inflation matrices, by applying the function in (12) to the estimated noises in (11),

13 $$\left\{ \begin{gathered} {{\boldsymbol{\bar {M}}}_1}=\left[ {\begin{array}{*{20}{c}} {\omega \left( {{{\bar {r}}_1}} \right)}&0& \cdots &0 \\ 0&{\omega \left( {{{\bar {r}}_2}} \right)}& \cdots &0 \\ \vdots & \vdots & \ddots & \vdots \\ 0&0& \cdots &{\omega \left( {{{\bar {r}}_n}} \right)} \end{array}} \right] \hfill \\ {{\boldsymbol{\bar {M}}}_2}=\left[ {\begin{array}{*{20}{c}} {\omega \left( {{{\bar {s}}_1}} \right)}&0& \cdots &0 \\ 0&{\omega \left( {{{\bar {s}}_2}} \right)}& \cdots &0 \\ \vdots & \vdots & \ddots & \vdots \\ 0&0& \cdots &{\omega \left( {{{\bar {s}}_m}} \right)} \end{array}} \right] \hfill \\ \end{gathered} \right.$$.

Introduce the following modified/inflated covariance matrices of the process and the measurement noises, using the covariance inflation matrices calculated in (13),

14 $$\left\{ \begin{gathered} {{\boldsymbol{\bar {W}}}_{k - 1}}=\boldsymbol{Q}{{\boldsymbol{\bar {M}}}_1}{\boldsymbol{Q}^{\mathrm{T}}} \hfill \\ {{\bar {{\boldsymbol{R}}}}_k}=\boldsymbol{S}{{\boldsymbol{\bar {M}}}_2}{\boldsymbol{S}^{\mathrm{T}}} \hfill \\ \end{gathered} \right.$$

In the current iteration of the proposed iterative algorithm, the standard Kalman filtering is done once again, but with the modified/inflated covariance matrices in (14) replacing the original ones; for an easy reference, it is repeated as follows,

15 $$\left\{ \begin{gathered} {{\boldsymbol{\hat {x}}}_{k\left| {k - 1} \right.}}={\boldsymbol{F}_{k - 1}}{{\boldsymbol{\hat {x}}}_{k - 1\left| {k - 1} \right.}} \hfill \\ {\boldsymbol{P}_{k\left| {k - 1} \right.}}={\boldsymbol{F}_{k - 1}}{\boldsymbol{P}_{k - 1\left| {k - 1} \right.}}\boldsymbol{F}_{{k - 1}}^{{\mathrm{T}}}+{{\boldsymbol{\bar {W}}}_{k - 1}} \hfill \\ {\boldsymbol{K}_k}={\boldsymbol{P}_{k\left| {k - 1} \right.}}\boldsymbol{H}_{k}^{{\mathrm{T}}}{\left( {{\boldsymbol{H}_k}{\boldsymbol{P}_{k - 1\left| {k - 1} \right.}}\boldsymbol{H}_{k}^{{\mathrm{T}}}+{{\bar {{\boldsymbol{R}}}}_k}} \right)^{ - 1}} \hfill \\ {{\boldsymbol{\hat {x}}}_{k\left| k \right.}}={{\boldsymbol{\hat {x}}}_{k\left| {k - 1} \right.}}+{\boldsymbol{K}_k}\left( {{\boldsymbol{y}_k} - {\boldsymbol{H}_k}{{\boldsymbol{\hat {x}}}_{k\left| {k - 1} \right.}}} \right) \hfill \\ {\boldsymbol{P}_{k\left| k \right.}}={\boldsymbol{P}_{k\left| {k - 1} \right.}} - {\boldsymbol{K}_k}{\boldsymbol{H}_k}{\boldsymbol{P}_{k\left| {k - 1} \right.}} \hfill \\ \end{gathered} \right.$$

Do the computations in (11), (13), (14) and (15) in order, one can modify the estimate obtained from the standard Kalman filter, with better robustness. One often needs to iterate the computations in (11)-(13)-(14)-(15), with the newest estimate in (15) as the initial value, namely ${\boldsymbol{\bar {x}}_k} \leftarrow {\boldsymbol{\hat {x}}_{k\left| k \right.}}$. This completes the introduction of the proposed algorithm. For easy reference, the proposed algorithm is summarized in Table 1.

**Table 1:** The operations at each epoch of the proposed algorithm.

Steps	Operations
[1]	Do the standard Kalman filtering as in (2).
[2]	Compute the estimates of the normalized noises according to (11).
[3]	Compute the covariance inflation matrices according to (13).
[4]	Modify/inflate the covariance matrices according to (14).
[5]	Do Kalman filtering according to (15).
[6]	If another iteration is needed, go to [2]; otherwise go to [7].
[7]	Terminate the algorithm at the current epoch and go to next epoch.

As readily shown in Table 1, the proposed method is simple and highly automated. While the performance of the proposed method, namely the filtering accuracy, is demonstrated by experiments in next section, the generality and flexibility are briefly explained here. Readily, if the measurement noise is believed to be normally distributed, one can let *ρ*_2_ in (9) be quadratic; and hence in the proposed method, only ***W***_*k*−1_ needs to be modified. The same goes for the process noise^38,39^. With the filter proposed here, one can even address the case with an arbitrary subset of heavy-tailed elements of the measurements and process noises, as long as this situation is believed to reflect the reality.

Computational Complexity: To explicitly evaluate the engineering implementability of the proposed method, the computational complexities of the Standard KF and the proposed HKF are compared, specifically focusing on the number of multiplication operations. Let n and m denote the dimensions of the state vector and measurement vector, respectively. In standard algorithmic complexity analysis, lower-order vector operations are typically omitted. All methods account for standard code-level optimizations commonly adopted in practice, such as intermediate matrix caching and exploiting matrix symmetry.

**Table 2:** Comparison of computational complexity between KF and HKF.

Filter	Approximate Number of Multiplications
KF	(2n^3^+n^2^) +(2n^2^m+2nm^2^+m^3^)
HKF	(2n^3^+n^2^)+(n^3^ + m^3^)/6 + N_iter_⋅(2n^2^m +2nm^2^ + m^3^+c_1_)

As detailed in Table 2, the proposed HKF fundamentally consists of a non-iterative initialization phase (prediction and Cholesky decomposition) followed by an iterative update loop. A key feature of the HKF is the dual-variance inflation. Consequently, within each iteration of the IRLS framework, the algorithm performs: (i) the core KF update, and (ii) the robust operations, including normalized noise estimation and variance-component reconstruction. The robust operations introduce an additional computational overhead per iteration, denoted as c_1_≈3n^3^+2m^3^+2n^2^+2m^2^+mn. Although the inclusion of process noise inflation introduces O(n^3^) terms into the iteration loop, the required number of iterations for convergence is typically very small (N_iter_≤5). The overall time complexity of the proposed HKF remains strictly bounded at O(N_iter_(n^3^ + m^3^)).

### Algorithm analysis and stability

The proposed robust Kalman filter is an IRLS-based iterative algorithm with a variance-component inflation strategy, which retains the recursive structure of the standard Kalman filter and achieves targeted robust processing for heavy-tailed process and measurement noises. This section analyzes the algorithm’s core performance characteristics from four key aspects to verify its theoretical stability and engineering implementability.

1. Convergence guarantee and local optimality: Since the adopted Huber loss function is convex, the IRLS iteration ensures the monotonic decrease of the objective function, which theoretically guarantees convergence to the unique global optimum.
2. Divergence and boundedness of the covariance matrix: The variance inflation function defined in this method is always greater than or equal to 1, so the adjusted covariance matrix remains strictly positive definite. This mathematically prevents singularity in the Kalman gain calculation and completely eliminates filter divergence.
3. Sensitivity to initialization: The proposed iterative method is initialized by the output of the standard Kalman filter. Since the initial value is in a high-confidence region and the optimization problem is convex, the algorithm is robust to initialization.
4. Stopping criterion: In practice, the iteration terminates when the norm difference of the state vector updates between two consecutive iterations is below a preset threshold, or when the maximum number of iterations is reached (e.g., 3 to 5 iterations as tested in the experiments of Sect. 3).

## Numerical examples

### Simulation

A target tracking problem is considered here, mainly to demonstrate the effectiveness of the proposed method^40^. The true kinematics of the target is assumed to be the following stochastic constant-velocity model^41^,

16 $$\left[ {\begin{array}{*{20}{c}} {{x_k}} \\ {{v_k}} \end{array}} \right]=\left[ {\begin{array}{*{20}{c}} 1&\tau \\ 0&1 \end{array}} \right]\left[ {\begin{array}{*{20}{c}} {{x_{k - 1}}} \\ {{v_{k - 1}}} \end{array}} \right]+\left[ {\begin{array}{*{20}{c}} {{\alpha _{k - 1}}} \\ {{\beta _{k - 1}}} \end{array}} \right]$$

where *x*_*k*_ and *v*_*k*_ denote the position and the velocity, respectively; true values *x*_0_=20 m and *v*_0_ = 2 are assigned; *τ* denotes the sampling interval, and let *τ* = 1; the noises ***α***_*k−1*_ and *β*_*k*−1_ are assumed independent with each other; their nominal standard deviations are assumed known, being 0.1 m and 0.2 m/s, respectively. However, the true distribution of the two noises are heavy-tailed ones with the following probability density function (PDF),

17 $$f\left( a \right)=\left( {1 - \Omega } \right)\left[ {\frac{1}{\sigma }{f_0}\left( {\frac{a}{\sigma }} \right)} \right]+\Omega \left[ {\frac{1}{{6\sigma }}{f_0}\left( {\frac{a}{{6\sigma }}} \right)} \right]$$

where *f*_0_ denotes the PDF of the standard normal distribution; the contamination ratio *Ω* is set to be 0.1; the nominal standard deviations *σ* in (17) are set to be the known nominal ones. We strictly set the standard deviation of the outlier component to 6 times the nominal standard deviation, so the variance of the outlier contamination component is 36 times the nominal variance, which fully simulates a severe heavy-tailed contaminated environment. The model in (17) is called a Gaussian mixture model, which is a special case of the contaminated normal model; its tails are heavier than that of the normal distribution with the nominal standard deviation.

The first element of the state vector, namely *x*_*k*_, is measured; and hence one has the following measurement model,

18 $${y_k}=\left[ {\begin{array}{*{20}{c}} 1&0 \end{array}} \right]\left[ {\begin{array}{*{20}{c}} {{x_k}} \\ {{v_k}} \end{array}} \right]+{\boldsymbol{\eta}_k}$$

where the nominal standard deviation of *e*_*k*_ is assumed known, being 1 m; the true PDF is also the model in (17). The time span of the simulation is 0 ≤ *k* ≤ 200.

The following three algorithms are employed for filtering with the state space model (16)/(18).

- KF: the standard Kalman filter;
- RKF: the robust Kalman filter^35^ with (6) as its loss function. RKF is the classical robust Kalman filter based on the Robust Bayesian Estimation framework. As the classic foundational M-M filtering scheme, it possesses the rare theoretical capability to simultaneously address the heavy-tailed non-normality of both process and measurement noises. It is selected as the primary baseline to ensure a strictly fair and rigorous evaluation, as both this classic RKF and the proposed HKF utilize the Iteratively Reweighted Least-Squares (IRLS) numerical architecture and share a comparable computational complexity.
- HKF: the heavy-tailed Kalman filter proposed in this contribution, with (9) as its loss function. HKF is the heavy-tailed Kalman filter proposed in this contribution. It is mathematically optimized from the classic RKF framework. While the RKF achieves dual-robustness by treating the predicted state estimation error as a proxy (inflating ***P***_*k|k−*1_), our HKF optimizes this structure by directly decomposing the error and specifically inflating the underlying process noise covariance (***W***_*k*−1_). This architectural optimization achieves higher-fidelity noise modeling.

All three filters are initialized with ${\boldsymbol{\hat {x}}_{0\left| 0 \right.}}=0$ and $${\boldsymbol{P}_{0\left| 0 \right.}}=\left[ {\begin{array}{*{20}{c}} {10000}&0 \\ 0&{100} \end{array}} \right]$$. The above nominal standard deviations are used to construct the covariance matrices of the process noise and the measurement noise, which are needed in all three filters.

**Fig. 1:**
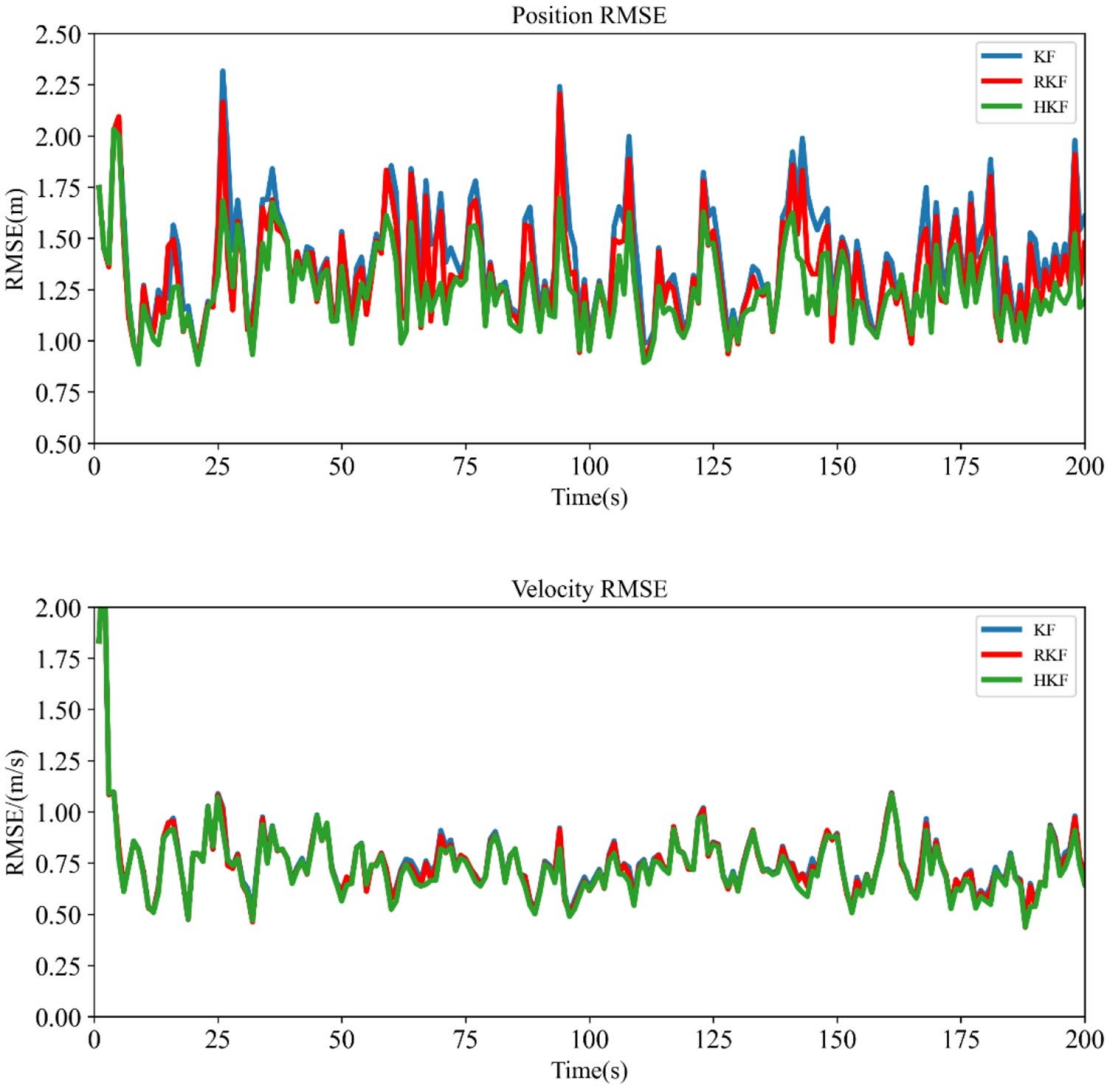
Root mean squared error of the filtering estimates at each epoch across 50 Monte Carlo runs for KF, RKF and HKF.

In order to evaluate the averaged performances of the competing methods, Monte Carlo experiments are conducted with totally 50 independent runs. The root mean squared errors (RMSE) and standard deviation (STD) of the filtering estimates at each epoch and across 50 Monte Carlo runs are shown in Fig. 1. Note that in RKF and HKF, besides the standard Kalman filtering operations, only one more iteration is included. The statistics of the filtering errors at all 200 epochs and in all 50 Monte Carlo runs are summarized in Table 3.

**Table 3:** Statistics of the filtering errors at all 200 epochs and in all 50 Monte Carlo runs for KF, RKF and HKF.

Error	Filter	Max	Min	Mean	STD	RMSE
Position errors (m)	KF	11.1079	−9.1782	0.0302	1.4200	1.4203
	RKF	10.8068	−8.9843	0.0242	1.3455	1.3457
	HKF	8.4945	−8.2627	0.0240	1.2627	**1.2629**
Velocity errors (m/s)	KF	7.5598	−7.5116	0.0050	0.7829	0.7829
	RKF	7.5598	−7.5115	0.0066	0.7745	0.7745
	HKF	7.5598	−7.5115	0.0054	0.7622	**0.7622**

From Fig. 1, the performance improvement of the proposed HKF to both KF and RKF is visible, especially for the position errors. This is further confirmed quantitatively by the statistics in Table 3. The performance improvements, in terms of RMSE decrease, from KF to RKF is 5.3% and 1.1% for positioning and velocimetry, respectively; while that from KF to the proposed HKF are 11.1% and 2.6%.

As shown in Fig. 1, while the proposed HKF exhibits a significant robust advantage in the directly measured position state (top), the performances of the three algorithms are highly similar in the velocity component (bottom). From the perspective of estimation theory, this similarity is determined by the observability of the system. In this CV model, velocity is an indirectly observed state, whose update relies on the Kalman gain matrix. This indirect inference mechanism inherently smooths the state corrections. Through the derivation of the Kalman gain matrix for velocity in the single-axis simulation, it is revealed that the velocity update depends on the cross-covariance between position and velocity. Since this cross-covariance is generally smaller than the position covariance, position measurement outliers have a relatively minor impact on the velocity update. Consequently, compared to the standard KF, the accuracy improvement introduced by the robust covariance inflation mechanism in the velocity component is relatively limited.

It is noted that the RKF is robust, at least to a certain extent, against the coexisting heavy-tailed non-normality of both the process and measurement noises. However, some earlier studies had conservative views about RKF. Yang et al.^42^, believe that, the filter system cannot properly separate the outliers in the process model and the measurements. Our new results here have revealed that, at least when the process model and the measurement model are both distorted in the way that both the process noise and the measurement noise are heavy-tailed, the M-estimation theory can still work as long as the distorted models (heavy-tailed distributions) still fall in the range of the small-deviation model^20^.

**Fig. 2:**
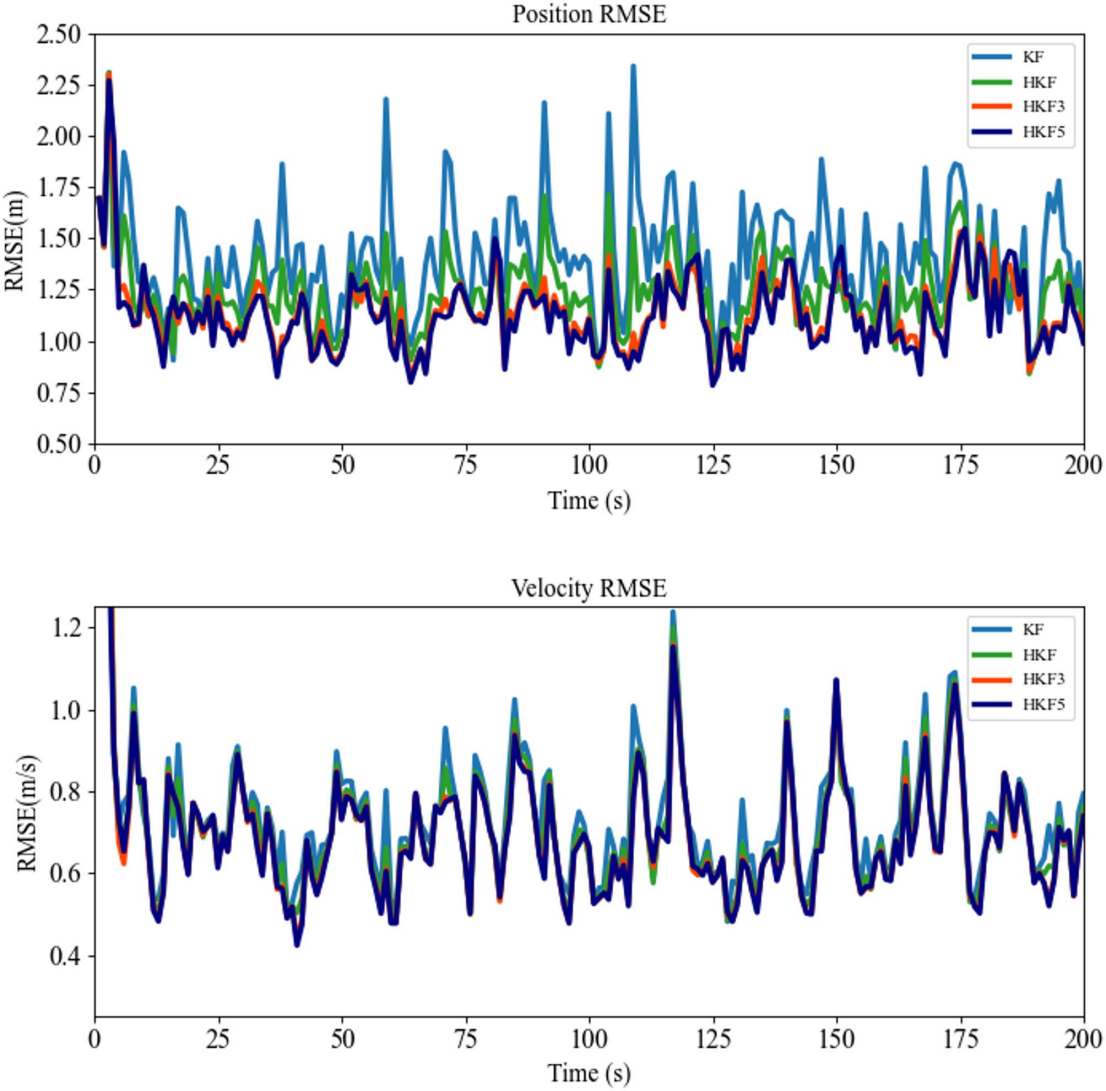
Root mean squared errors of the filtering estimates at each epoch across 50 Monte Carlo runs, for KF, and HKF with different numbers of iterations.

The possible improvements brought by more iterations in the proposed HKF are also checked with additional 50 Monte Carlo runs in this experiments. The root mean squared errors of the filtering estimates of HKF with different numbers of iterations are shown in Fig. 2. In this figure, the number following the term “HKF” denotes the numbers of the iterations included. The statistics of the filtering errors at all 200 epochs and in all 50 Monte Carlo runs are summarized in Table 4. From Fig. 2, the additional improvements brought by more iterations are visible. The improvements become marginal with the increasing numbers of iterations. These findings are also validated quantitatively by the statistics in Table 4. The performance improvements for positioning, in terms of RMSE decreases, from KF to HKF with 1, 3, and 5 iterations, are 12.3%, 19.1% and 20.0%, respectively. The values for velocimetry are 3.2%, 4.9% and 5.2%.

**Table 4:** Statistics of the filtering errors at all 200 epochs and in all 50 Monte Carlo runs for KF, and HKF with different numbers of iterations.

Error	Filter	Max	Min	Mean	STD	RMSE
Position errors (m)	KF	11.5828	−10.2354	−0.0209	1.4253	1.4254
	HKF1	8.0282	−9.0351	−0.0135	1.2496	1.2497
	HKF3	7.9190	−9.0352	−0.0065	1.1518	1.1519
	HKF5	7.8481	−10.0076	−0.0058	1.1405	**1.1405**
Velocity errors (m/s)	KF	9.5940	−6.8115	−0.0108	0.7730	0.7731
	HKF1	9.5940	−6.8115	−0.0112	0.7483	0.7484
	HKF3	9.5943	−6.8132	−0.0115	0.7351	0.7352
	HKF5	9.5943	−6.8121	−0.0117	0.7327	**0.7328**

**Fig. 3:**
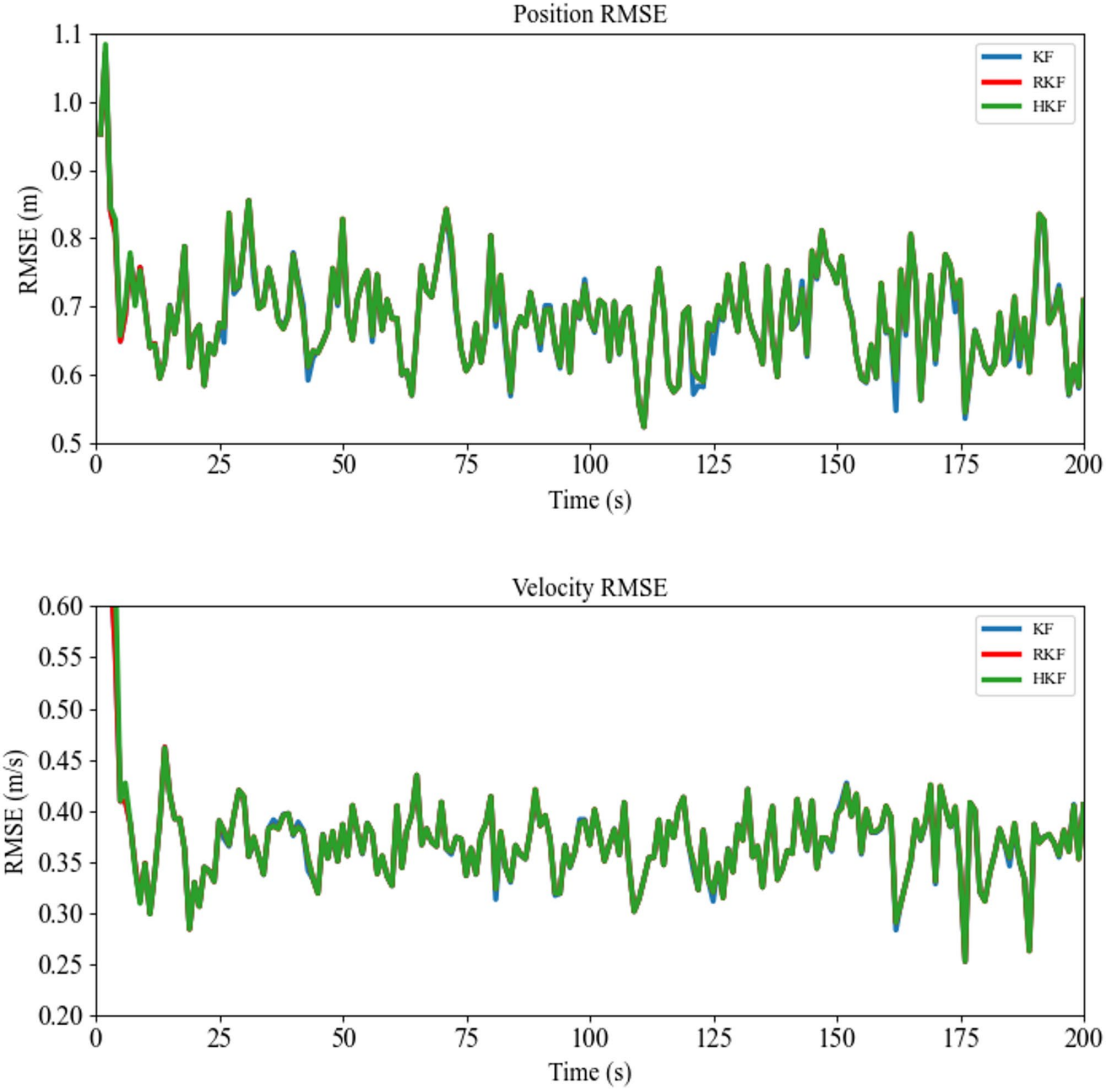
Root mean squared error of the filtering estimates at each epoch across 50 Monte Carlo runs for KF, RKF and HKF, when the real and nominal distributions coincide.

Besides the increased computational time (which is trivial, given the computing-power for modern systems), another price that must be paid for the robustness is the efficiency loss (variance increase) when the real distributions are indeed the assumed normal ones. Fortunately this price is low, according to the M-estimation theory. In this simulation, the efficiency loss is also checked. The following Fig. 3; Table 5 are counterparts of Fig. 1; Table 3, respectively, when the real and the nominal distributions coincide. From Fig. 3, the error plots of the three competing method are almost indistinguishable. Quantitatively from Table 5, the standard KF performs the best, as expected; however, the performance decreases in terms of RMSE increase of the two robust methods (RKF/HKF) are only 0.33%/0.38% for positioning and 0.10%/0.82% for velocimetry. Under this ideal situation, the conventional RKF performs better than the proposed HKF. One can say that the price paid by the proposed HKF for robustness is higher than that of the conventional RKF. However, considering the almost indistinguishability of the three methods, this higher price is trivial. From Table 5 to Table 4 or 2, the performances of all three methods degrade; this is rational, because the data qualities become worse. However, the performance degradations of the robust methods are much slighter; and this is exactly what robust methods aim to do.

Figure 3 presents an ideal simulation scenario where the true noise strictly follows a nominal Gaussian distribution without any heavy-tailed contamination. Under such conditions, the standard KF is mathematically the optimal Best Linear Unbiased Estimator. The similar performance of the proposed HKF—with an extremely marginal efficiency loss—precisely demonstrates its statistical safety. By adopting the classic Huber threshold parameter *c* = 1.345, the HKF is theoretically guaranteed to maintain 95% asymptotic efficiency relative to the standard KF. This ensures that the robust mechanism does not conservatively sacrifice nominal accuracy when robust processing is unneeded.

**Table 5:** Statistics of the filtering error at all 200 epochs and in all 50 Monte Carlo runs for KF, RKF and HKF, when the real and nominal distributions coincide.

Error	Filter	Max	Min	Mean	STD	RMSE
Position errors (m)	KF	2.7235	−2.7825	−0.0271	0.6880	**0.6885**
	RKF	2.7235	−2.7825	−0.0271	0.6903	0.6908
	HKF	2.7682	−2.7821	−0.0277	0.6906	0.6911
Velocity errors (m)	KF	3.2937	−3.3920	−0.0133	0.4038	**0.4041**
	RKF	3.2937	−3.3920	−0.0133	0.4043	0.4045
	HKF	3.4045	−3.4237	−0.0144	0.4071	0.4074

**Fig. 4:**
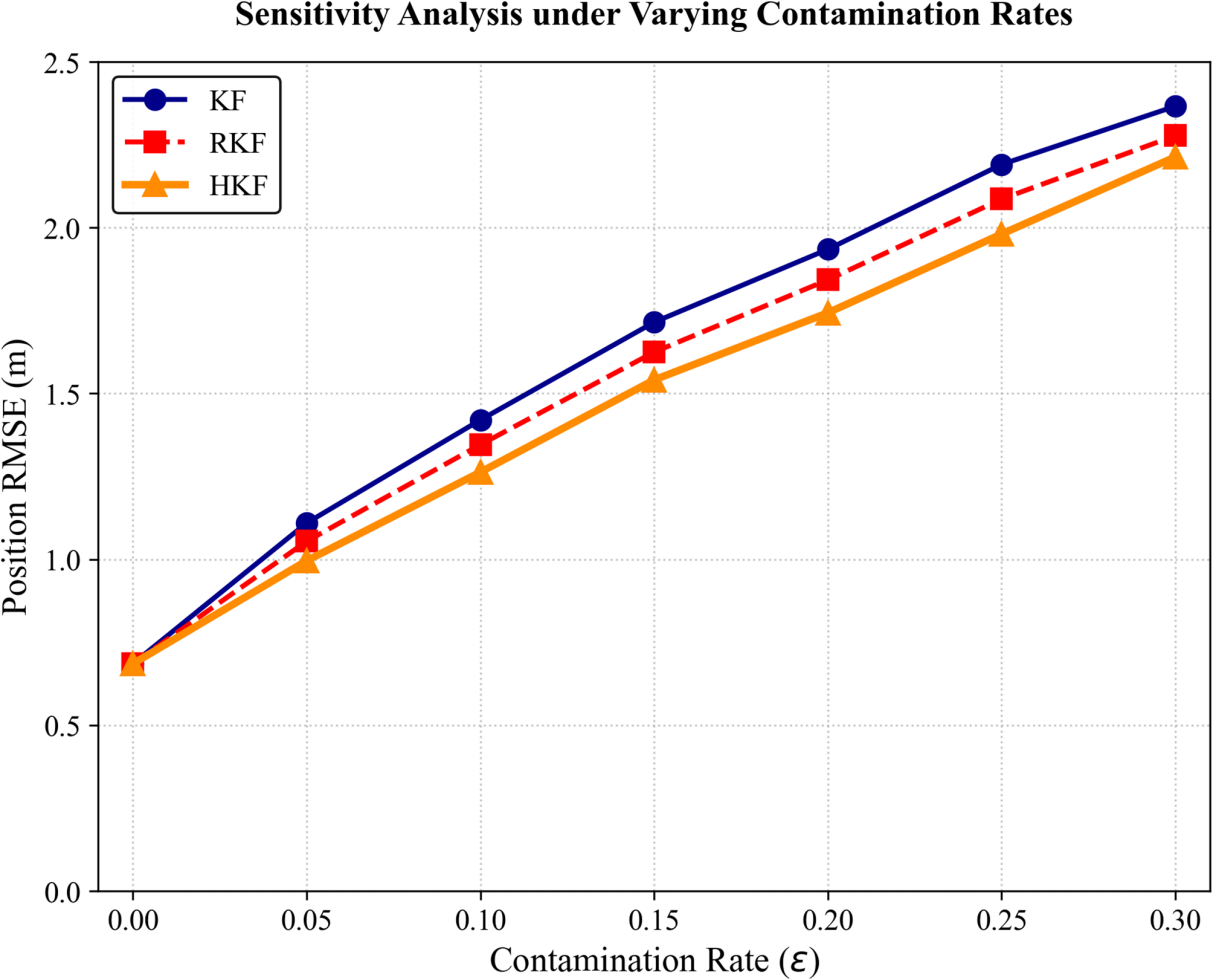
Sensitivity analysis under varying contamination rates.

To comprehensively evaluate the robust performance of the algorithm, this study tests its sensitivity under different contamination rates. The experimental results presented in Fig. 4 show a typical smooth evolutionary trend that conforms to the characteristics of the Gaussian Mixture Model.

Under the pure nominal Gaussian distribution, the RMSEs of HKF, RKF, and the standard KF are highly consistent and converge to approximately 0.69 m. This confirms that the proposed robust mechanism maintains strict statistical safety, ensuring that no severe nominal performance degradation is triggered when robust processing is unneeded.

With the increase of the contamination rate, the equivalent variance of the system mixed noise expands linearly, leading to a square root sublinear growth trend of the RMSE for all three algorithms, which is consistent with theoretical expectations. In this process, the standard KF does not diverge significantly due to Monte Carlo averaging and the zero-mean noise characteristics, but its estimation error is significantly higher. In contrast, the proposed HKF algorithm relies on the optimized truncated weight function to effectively isolate and downweight abnormal observations over the entire wide contamination range from 0.05 to 0.3. As reflected in Fig. 4, the positioning error curve of HKF remains consistently below those of KF and RKF across all experimental epochs, and the data show that regardless of the system’s contamination level, HKF can stably suppress the RMSE to the optimal level and achieve a stable accuracy improvement of about 10% compared with the standard KF.

This sensitivity analysis fully demonstrates that the HKF algorithm not only satisfies the theoretical small deviation assumption, but also provides stable and consistent robust gains across the entire continuous spectrum of contamination ratio evolution, showing engineering reliability.

### Real data processing

This is a dynamic car-borne integrated GNSS Single Point Positioning (SPP) and INS experiment. Data were collected on the Nanhu campus of China University of Mining and Technology. The time span is 180 min. GNSS data were collected with a Trimble R10 receiver with 1 Hz sampling rate; while the INS data were collected with a Inertial Measurement Unit (IMU) CPT with 100-Hz sampling rate. Loose coupling mode is employed for the GNSS/INS integration^43^. The positions and velocities are calculated only using BeiDou Navigation Satellite System (BDS) pseudoranges and BDS Doppler observables. These positions and velocities are used as measurements for the state space model. The data processings are done with the open-source software GINav^44^. The postprocessing or smoothing estimates following the integrated Real-Time Kinematic (RTK) and INS tight coupling mode provided by Inertial-Explorer software are chosen as references to compute the filtering errors.

**Fig. 5:**
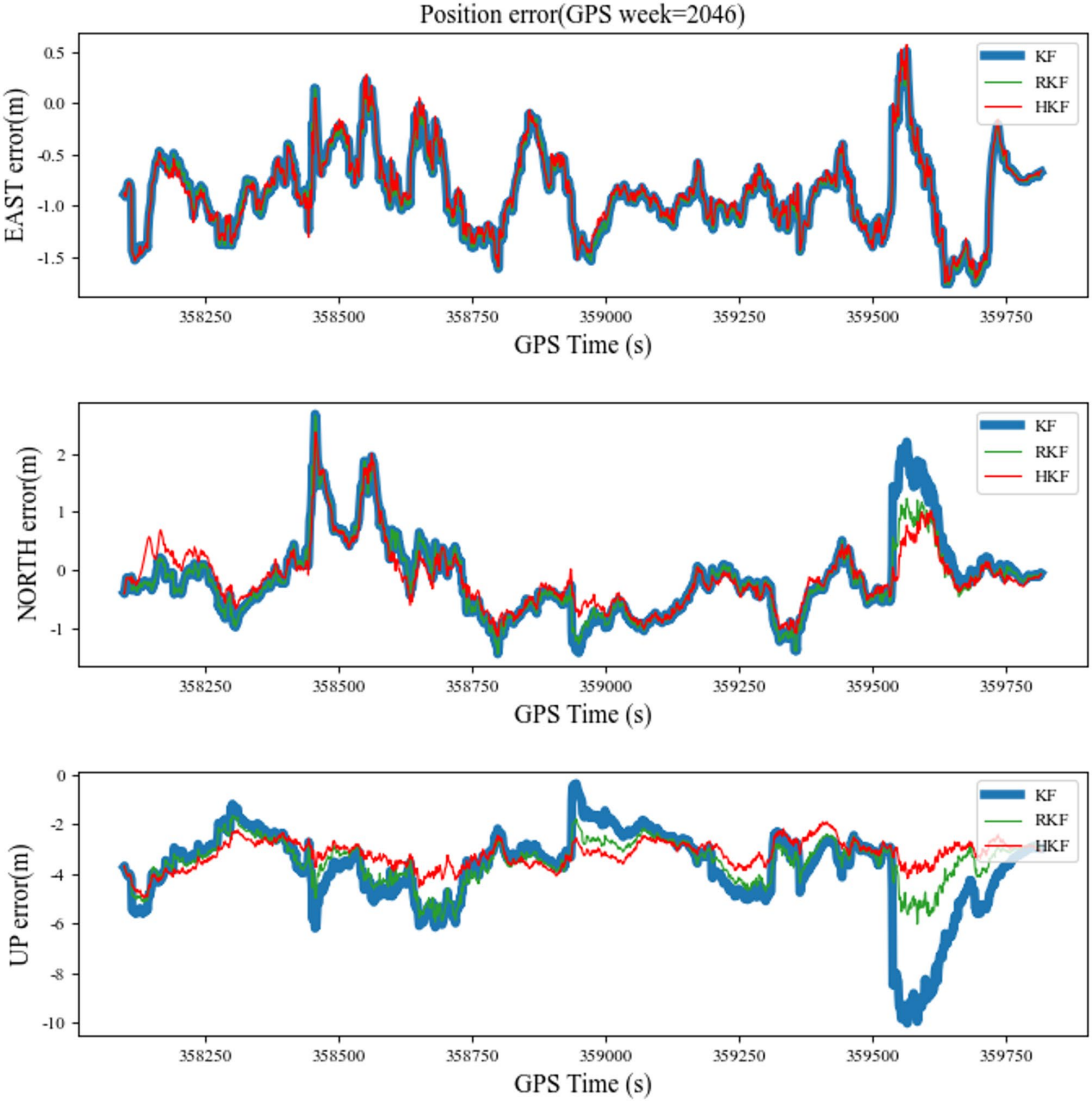
Positioning errors in the GNSS/INS experiments.

**Fig. 6:**
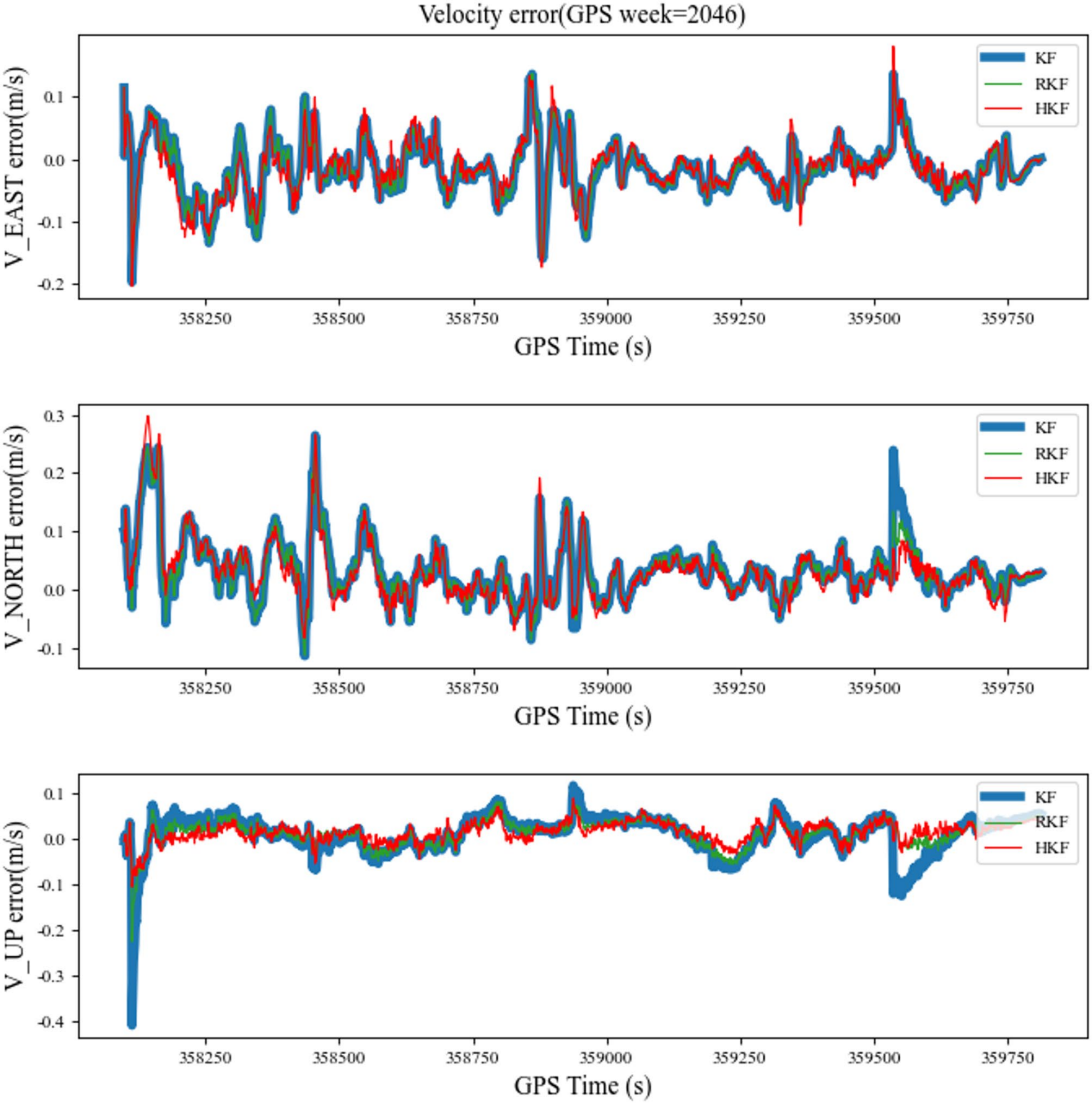
Velocimetry errors in the GNSS/INS experiments.

**Fig. 7:**
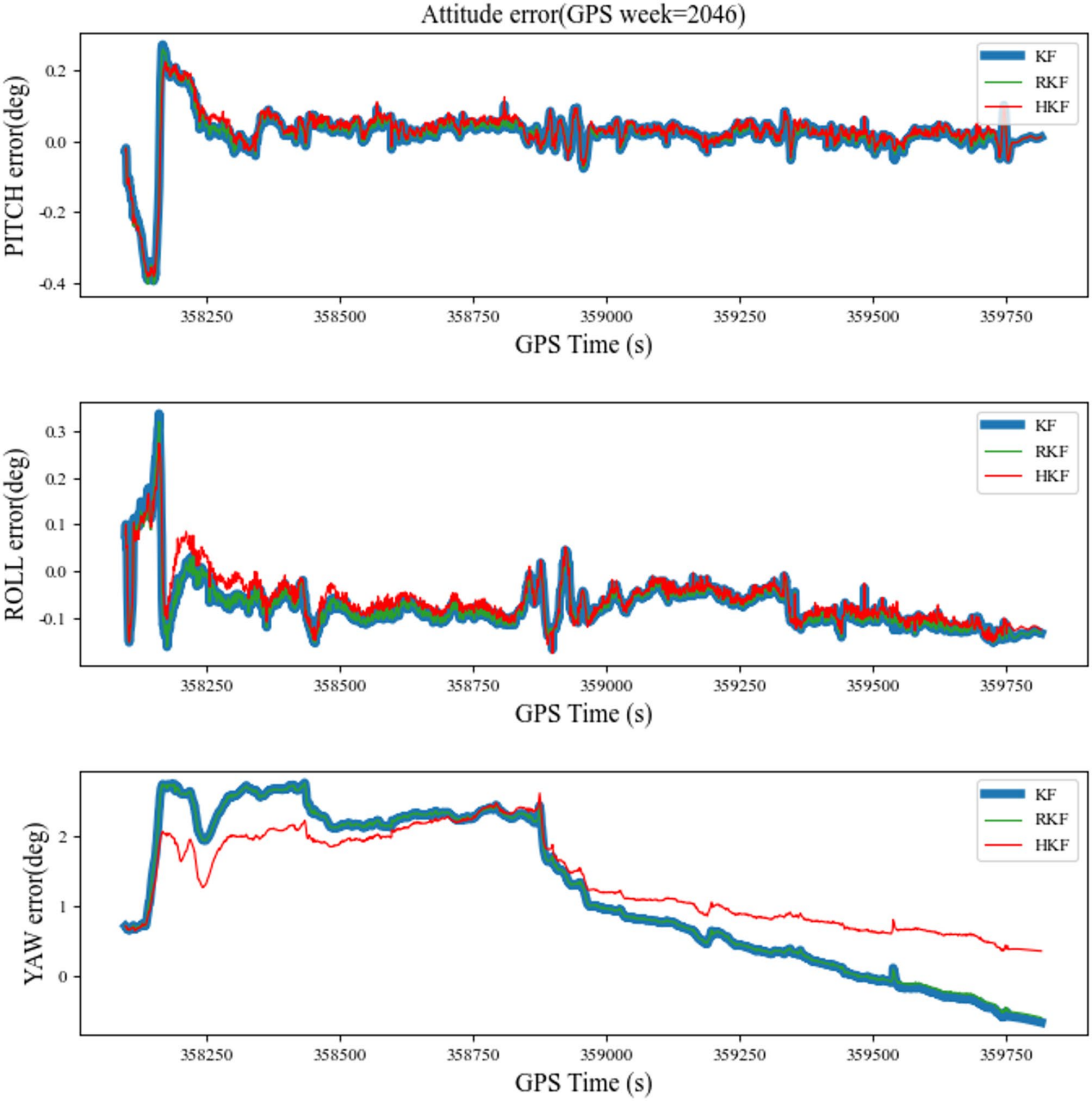
Attitude errors in the GNSS/INS experiments.

The filtering errors at different epochs for positioning, velocimetry and attitude determination are plotted in Figs. 5, 6 and 7, respectively. The statistics of the errors are summarized in Tables 6, 7 and 8. The findings are in order.

[1] In general the navigation performance is improved with either the conventional RKF or the proposed HKF, when compared to traditional KF. This tells that in this experiment at least for some noise components and at some epochs, the distributions are heavy-tailed. HKF performs better than RKF. This confirms the effectiveness of the proposed method. To be quantitative, from Tables 6, 7 and 8, the positioning performance improvements of the proposed HKF compared to KF, in terms of RMSE, are 1.6%, 21.0% and 22% along east, north and upward directions, respectively. The velocimetry performance improvements are 0.7%, 2.3% and 41.6%. The attitude determination performance improvements are − 4.2%, 11.1% and 6.8%. In the real-world GNSS/INS experiment, the East position error (Fig. 5 top) exhibits highly similar RMSEs across all methods. Unlike simulated global contamination, real-world measurement anomalies often exhibit spatial heterogeneity. As analyzed in Sect. 3.3, the highly overlapping confidence intervals indicate that the local measurement noise in the East direction along this specific trajectory was dominantly Gaussian. Consequently, the robust mechanisms were minimally triggered along this specific axis, resulting in very limited accuracy improvements from the robust algorithms. This stands in contrast to the North and Upward directions, where non-Gaussian anomalies were more frequent.

[2] Improvements brought by including more iterations in HKF is insignificant; the results presented here are all those from the HKF with only one iteration.

[3] For some navigation variables such as east position and horizontal velocities, the improvements are marginal.

[4] As shown in Table 7; Fig. 6, both robust methods are slightly inferior to the standard KF in estimating the pitch angle, whereas all three methods perform comparably for the other two attitude components. This slight degradation can occur when the actual noise distribution is sufficiently close to a Gaussian one; moreover, even under heavy-tailed non-Gaussian conditions, robust methods may still underperform in specific scenarios. Furthermore, since the pitch angle cannot be directly observed by GNSS, its error inherently accumulates within the INS over time and heavily relies on the GNSS-derived north velocity for correction. Consequently, when the robust filtering mechanism down-weights the north velocity updates to suppress outliers, it inadvertently weaken or block the effective correction information required for attitude estimation, ultimately leading to the accumulation of pitc

**Table 6:** Statistics of positioning errors in the GNSS/INS experiment.

Errors	Filter	Max	Min	Mean	STD	RMSE
East (m)	KF	0.5077	−1.7624	−0.8732	0.3879	0.9555
	RKF	0.5296	−1.8200	−0.8877	0.3672	0.9606
	HKF	0.5698	−1.7465	−0.8563	0.3879	**0.9401**
North (m)	KF	2.6819	−1.4502	−0.1200	0.7107	0.7208
	RKF	2.6538	−1.4521	−0.1622	0.6257	0.6464
	HKF	2.3682	−1.1471	−0.1151	0.5577	**0.5695**
Upward (m)	KF	−0.3655	−10.0098	−3.8198	1.6615	4.1655
	RKF	−1.6548	−6.0071	−3.5149	0.8693	3.6208
	HKF	−1.9067	−4.9500	−3.2037	0.5407	**3.2490**

**Table 7:** Statistics of velocimetry errors in the GNSS/INS experiment.

Errors	Filter	Max	Min	Mean	STD	RMSE
East (m/s)	KF	0.1357	−0.1968	−0.0154	0.0417	0.0445
	RKF	0.1394	−0.1969	−0.0155	0.0418	0.0445
	HKF	0.1798	−0.2035	−0.0150	0.0416	**0.0442**
North (m/s)	KF	0.2648	−0.1139	0.0327	0.0514	0.0609
	RKF	0.2610	−0.1127	0.0321	0.0514	0.0596
	HKF	0.2983	−0.0831	0.0302	0.0512	**0.0595**
Upward (m/s)	KF	0.1156	−0.4089	0.0088	0.0426	0.0435
	RKF	0.0797	−0.2254	0.0110	0.0282	0.0302
	HKF	0.0876	−0.1058	0.0133	0.0217	**0.0254**

**Table 8:** Statistics of attitude errors in the GNSS/INS experiment.

Errors	Filter	Max	Min	Mean	STD	RMSE
Pitch (deg)	KF	0.2714	−0.3938	0.0198	0.0705	**0.0732**
	RKF	0.2591	−0.4033	0.0202	0.0719	0.0764
	HKF	0.2225	−0.3820	0.0262	0.0718	0.0764
Roll (deg)	KF	0.3358	−0.1657	−0.0709	0.0556	0.0901
	RKF	0.3190	−0.1659	−0.0714	0.0530	0.0890
	HKF	0.2727	−0.1741	−0.0584	0.0547	**0.0801**
Yaw (deg)	KF	2.7585	−0.6673	1.1923	1.1067	1.6268
	RKF	2.7514	−0.5964	1.1964	1.0903	1.6261
	HKF	2.6101	0.3535	1.1652	0.9590	**1.5160**

### Statistical significance

To quantitatively verify the statistical significance of the root mean square error improvements achieved by the proposed HKF algorithm over the standard KF and the conventional RKF, and distinguish such valid performance gains from random experimental fluctuations, two types of statistical analysis are conducted for the two experiments in this study. Paired two-tailed t-tests at the 95% confidence level with a significance level of 0.05 are implemented for the Monte Carlo simulation experiment in Sect. 3.1, and 95% confidence interval analysis is carried out for the real GNSS/INS experiment in Sect. 3.2, aiming to fully validate the reliability of the algorithm’s performance advantages.

For the Monte Carlo simulation experiment, paired two-tailed t-tests are performed on the positioning and velocimetry RMSE series of KF, RKF and HKF under the heavy-tailed noise scenario, with the null hypothesis that no statistically significant difference exists between the filtering errors of the two compared algorithms. In the velocimetry dimension, the overall RMSE values of the standard KF, conventional RKF and the proposed HKF are 0.7825 m/s, 0.7742 m/s and 0.7617 m/s respectively. The performance improvement of the HKF algorithm over the standard KF is of highly statistical significance with the p-value of 5.44e-26, and the RKF algorithm also passes the statistical significance test for its performance improvement over the KF with the p-value of 2.01e-25. In the positioning dimension, the overall RMSE values of the standard KF, conventional RKF and the proposed HKF are 1.4213 m,1.34564 m and 1.2626 m respectively. The performance improvement of HKF over KF is of highly statistical significance with the p-value of 1.33e-36, and the improvement of RKF over KF also exhibits statistical significance with the p-value of 1.79e-27. All p-values of the pairwise comparison groups are far less than the 0.05 significance threshold, which fully demonstrates that the performance improvements of the proposed HKF algorithm in the simulation experiment are not caused by random experimental fluctuations but possess stable and reproducible statistical significance, effectively verifying the robustness advantage of the HKF algorithm in heavy-tailed noise scenarios.

For the real GNSS/INS experiment, aiming at the marginal 1.6% RMSE improvement in the east direction, the 95% confidence intervals of the filtering errors for the standard KF and the proposed HKF are calculated as 0.864 to 0.899 and 0.850 to 0.884 respectively, with a substantial overlap observed between the two intervals. This overlapping characteristic indicates that the practical filtering performance of the two algorithms is extremely close in the east direction of the real experiment. According to robust estimation theory, the standard KF is a linear unbiased optimal estimator under pure Gaussian noise conditions. The high overlap of the two confidence intervals suggests that the actual observation noise in the east direction data of this real test is more consistent with the nominal Gaussian distribution assumption, lacking sufficient and severe non-Gaussian outliers to trigger the significant advantage of the robust mechanism of the HKF algorithm, which objectively limits the room for the robust estimation to exert its effect. Notably, this result further verifies a crucial property of the proposed HKF algorithm. In the mild environment close to Gaussian distribution where robust processing is almost unnecessary, the HKF algorithm still maintains robust statistical safety. It can perfectly degenerate and approach the optimal estimation accuracy of the standard KF without causing the degradation of nominal performance due to overly conservative robust downweighting operations. This property ensures the reliability of the HKF algorithm in both Gaussian and heavy-tailed non-Gaussian noise scenarios for both simulation and real application scenarios.

## Concluding remarks

Despite its beautiful theoretical properties^45^ and its wide practical applications^2,3^, Kalman filtering is not robust against heavy-tailed distributions of the process and/or measurement noises. Modifications can be made, based on M-estimation theory^19,20^, to make the Kalman filtering robust. The proposed method inherits the merits of the standard Kalman filtering, namely having the same simple algorithmic structure readily permitting real-time implementations. The additional merit of the proposed robust method is that it is more targeted specifically towards the suspiciously heavy-tailed noises. The process and measurement noises are treated in a robust way; while the other error sources, namely the previous-epoch filtering state errors deemed to be normally distributed, are treated in a least-squares way. This more-targeted M-estimation based robust Kalman filter scheme implies higher-fidelity modeling. So, it should not be surprising to expect improved filtering performance with the proposed method.

Simulation studies of a simple tracking problem demonstrated the performance of the new method. Compared to the standard Kalman filtering, the improvements of the positioning performance in terms of root mean squared errors of the filtering estimates can be 11.1%. The percent can be increased to 20.0% if the computational resource allows several, say 5, iterations of the operations involved in the proposed method. Moreover, the improvement over the previous robust Kalman filter schemes is 5.3% for this simulated case study.

The performance of the proposed method is also checked through real data analysis of a GNSS/INS integration experiment. The positioning performance in terms of root mean squared filtering errors can be improved; from the standard Kalman filtering to the proposed method, with the improvements of 1.6%, 21.0% and 22% along east, north and upward directions, respectively.

In practical navigation applications, obtaining an exact mathematical model of error distributions is often impossible. The conventional Gaussian assumption is frequently an oversimplification, making the occurrence of heavy-tailed process and measurement noises practically inevitable. To mitigate the severe performance degradation caused by these non-nominal noises, robust structural modifications to the standard Kalman filter are essential. The proposed HKF serves as a practical upgrade. It is suggested to add a small module to existing software, to implement the robust modification proposed in this work.

Despite its effectiveness, the proposed method still has certain limitations. The IRLS iteration results in additional computational cost compared with the standard Kalman filter, and the method is mainly applicable to small-deviation model assumptions; moreover, the fixed threshold parameter *c* = 1.345 adopted in the Huber loss may lead to performance degradation in non-stationary environments. Future research will focus on constructing high-breakdown-point robust methods to suppress extreme outliers, designing adaptive strategies to dynamically tune robust parameters, extending the proposed framework to nonlinear filters such as the Extended Kalman Filter (EKF) and the Unscented Kalman Filter (UKF), and applying the method to more complex navigation scenarios with sparse or missing observations.

## Data Availability

The datasets used and analysed during the current study are available in the GINav repository, https://github.com/kaichen686/GINav/tree/main/data. Specifically, the data\_cpt.7z dataset (included in the repository as part of the shared data resources) was utilized for the study.

## Appendix A. Derivation of the solution to the problem of minimizing (9)

Equations (8) and (9) are repeated as follows,

19 $$\left\{ \begin{gathered} {\boldsymbol{l}_1}={\boldsymbol{L}_1}{\boldsymbol{x}_k}+{\boldsymbol{n}}+\boldsymbol{r} \hfill \\ {\boldsymbol{l}_2}={\boldsymbol{L}_2}{\boldsymbol{x}_k}+\boldsymbol{s} \hfill \\ \end{gathered} \right.$$

20 $$\phi ={\rho _1}\left( \boldsymbol{r} \right)+{\rho _2}\left( \boldsymbol{s} \right)+\frac{1}{2}{{\boldsymbol{n}}^{\mathrm{T}}}{\boldsymbol{U}^{ - 1}}{\boldsymbol{n}}+\boldsymbol{\boldsymbol{\uplambda}}_{1}^{{\mathrm{T}}}\left( {{\boldsymbol{l}_1} - {\boldsymbol{L}_1}{\boldsymbol{x}_k} - {\boldsymbol{n}} - \boldsymbol{r}} \right)+\boldsymbol{\boldsymbol{\uplambda}}_{2}^{{\mathrm{T}}}\left( {{\boldsymbol{l}_2} - {\boldsymbol{L}_2}{\boldsymbol{x}_k} - \boldsymbol{s}} \right)$$

The following matrices are adopted here for use in the derivation:

21 $$\left\{ \begin{gathered} {\boldsymbol{M}_1}=\left[ {\begin{array}{*{20}{c}} {\omega \left( {{r_1}} \right)}&0& \cdots &0 \\ 0&{\omega \left( {{r_2}} \right)}& \cdots &0 \\ \vdots & \vdots & \ddots & \vdots \\ 0&0& \cdots &{\omega \left( {{r_n}} \right)} \end{array}} \right] \hfill \\ {\boldsymbol{M}_2}=\left[ {\begin{array}{*{20}{c}} {\omega \left( {{s_1}} \right)}&0& \cdots &0 \\ 0&{\omega \left( {{s_2}} \right)}& \cdots &0 \\ \vdots & \vdots & \ddots & \vdots \\ 0&0& \cdots &{\omega \left( {{s_m}} \right)} \end{array}} \right] \hfill \\ \end{gathered} \right.$$

According to the necessary condition of the saddle point of (20), one has the following.

22 $$\left\{ \begin{gathered} \frac{{\partial \phi }}{{\partial \boldsymbol{r}}}=0 \Leftrightarrow \boldsymbol{r}={\boldsymbol{M}_1}{\boldsymbol{\boldsymbol{\uplambda}}_1} \hfill \\ \frac{{\partial \phi }}{{\partial \boldsymbol{s}}}=0 \Leftrightarrow \boldsymbol{s}={\boldsymbol{M}_2}{\boldsymbol{\boldsymbol{\uplambda}}_2} \hfill \\ \end{gathered} \right.$$

Insert (22) into (19), one has the following:

23 $$\left\{ \begin{gathered} {\boldsymbol{\boldsymbol{\uplambda}}_1}={\left( {\boldsymbol{U}+{\boldsymbol{M}_1}} \right)^{ - 1}}\left( {{\boldsymbol{l}_1} - {\boldsymbol{L}_1}{\boldsymbol{x}_k}} \right) \hfill \\ {\boldsymbol{\boldsymbol{\uplambda}}_2}=\boldsymbol{M}_{2}^{{ - 1}}\left( {{\boldsymbol{l}_2} - {\boldsymbol{L}_2}{\boldsymbol{x}_k}} \right) \hfill \\ \end{gathered} \right.$$

One also has the following necessary condition:

24 $$\frac{{\partial \phi }}{{\partial {\boldsymbol{x}_k}}}=0 \Leftrightarrow \boldsymbol{L}_{1}^{{\mathrm{T}}}{\boldsymbol{\boldsymbol{\uplambda}}_1}+\boldsymbol{L}_{2}^{{\mathrm{T}}}{\boldsymbol{\boldsymbol{\uplambda}}_2}=0$$

Insert (23) into (24), one has the following:

25 $${\boldsymbol{\hat {x}}_{k\left| k \right.}}={\left[ {\boldsymbol{L}_{1}^{{\mathrm{T}}}{{\left( {\boldsymbol{U}+{\boldsymbol{M}_1}} \right)}^{ - 1}}{\boldsymbol{L}_1}+\boldsymbol{L}_{2}^{{\mathrm{T}}}\boldsymbol{M}_{2}^{{ - 1}}{\boldsymbol{L}_2}} \right]^{ - 1}}\left[ {\boldsymbol{L}_{1}^{{\mathrm{T}}}{{\left( {\boldsymbol{U}+{\boldsymbol{M}_1}} \right)}^{ - 1}}{\boldsymbol{l}_1}+\boldsymbol{L}_{2}^{{\mathrm{T}}}\boldsymbol{M}_{2}^{{ - 1}}{\boldsymbol{l}_2}} \right]={\boldsymbol{\varTheta}^{ - 1}}\boldsymbol{\boldsymbol{\uptheta}}$$

Considering $$\left\{ \begin{gathered} {\boldsymbol{l}_1}={\boldsymbol{Q}^{ - 1}}{{\boldsymbol{\hat {x}}}_{k\left| {k - 1} \right.}} \hfill \\ {\boldsymbol{l}_2}={\boldsymbol{S}^{ - 1}}{\boldsymbol{y}_k} \hfill \\ \end{gathered} \right.$$, $$\left\{ \begin{gathered} {\boldsymbol{L}_1}={\boldsymbol{Q}^{ - 1}} \hfill \\ {\boldsymbol{L}_2}={\boldsymbol{S}^{ - 1}}{\boldsymbol{H}_k} \hfill \\ \end{gathered} \right.$$, and $\boldsymbol{U}={\boldsymbol{Q}^{ - 1}}{\boldsymbol{F}_{k - 1}}{\boldsymbol{P}_{k - 1\left| {k - 1} \right.}}\boldsymbol{F}_{{k - 1}}^{{\mathrm{T}}}{\boldsymbol{Q}^{ - {\mathrm{T}}}}$ in (25), one has the following, after some simple algebraic operations.

26 $$\left\{ \begin{gathered} \boldsymbol{\varTheta}={\left( {{\boldsymbol{F}_{k - 1}}{\boldsymbol{P}_{k - 1\left| {k - 1} \right.}}\boldsymbol{F}_{{k - 1}}^{{\mathrm{T}}}+\boldsymbol{Q}{\boldsymbol{M}_1}{\boldsymbol{Q}^{\mathrm{T}}}} \right)^{ - 1}}+\boldsymbol{H}_{k}^{{\mathrm{T}}}{\left( {\boldsymbol{S}{\boldsymbol{M}_2}{\boldsymbol{S}^{\mathrm{T}}}} \right)^{ - 1}}{\boldsymbol{H}_k} \hfill \\ \boldsymbol{\boldsymbol{\uptheta}}={\left( {{\boldsymbol{F}_{k - 1}}{\boldsymbol{P}_{k - 1\left| {k - 1} \right.}}\boldsymbol{F}_{{k - 1}}^{{\mathrm{T}}}+\boldsymbol{Q}{\boldsymbol{M}_1}{\boldsymbol{Q}^{\mathrm{T}}}} \right)^{ - 1}}{{\boldsymbol{\hat {x}}}_{k\left| {k - 1} \right.}}+\boldsymbol{H}_{k}^{{\mathrm{T}}}{\left( {\boldsymbol{S}{\boldsymbol{M}_2}{\boldsymbol{S}^{\mathrm{T}}}} \right)^{ - 1}}{\boldsymbol{y}_k} \hfill \\ \end{gathered} \right.$$

Insert (26) into (25), then insert (25) into (23), and then insert (23) into (22), one obtains the expressions in (11). Make the approximation **A1**: treating ***M***_1_ and ***M***_2_ as constants, rather than variables depending on ***x***_*k*_. Note that since the Huber loss is strictly convex, the optimization problem possesses a unique global optimum. This approximation ensures that the IRLS algorithm converges monotonically to this solution without the risk of local optima. Meanwhile, due to the mathematical symmetry of the Huber loss, treating ***M***_1_ and ***M***_2_ as constants within the iteration does not introduce systematic bias or destroy the unbiasedness of the state estimation.

Then the estimate in (25), with (26), is the least-squares estimate of a measurement model similar to that in (4), shown as follows.

27 $$\left[ {\begin{array}{*{20}{c}} {{{\boldsymbol{\hat {x}}}_{k\left| {k - 1} \right.}}} \\ {{\boldsymbol{y}_k}} \end{array}} \right]=\left[ {\begin{array}{*{20}{c}} {{\boldsymbol{I}_n}} \\ {{\boldsymbol{H}_k}} \end{array}} \right]{\boldsymbol{x}_k}+\left[ {\begin{array}{*{20}{c}} {{\boldsymbol{\boldsymbol{\upvarepsilon}}_k}} \\ {{\boldsymbol{\eta}_k}} \end{array}} \right]{\text{, with }}\operatorname{cov} \left( {\left[ {\begin{array}{*{20}{c}} {{\boldsymbol{\boldsymbol{\upvarepsilon}}_k}} \\ {{\boldsymbol{\eta}_k}} \end{array}} \right]} \right)=\left[ {\begin{array}{*{20}{c}} {{\boldsymbol{F}_{k - 1}}{\boldsymbol{P}_{k - 1\left| {k - 1} \right.}}\boldsymbol{F}_{{k - 1}}^{{\mathrm{T}}}+\boldsymbol{Q}{\boldsymbol{M}_1}{\boldsymbol{Q}^{\mathrm{T}}}}&0 \\ 0&{\boldsymbol{S}{\boldsymbol{M}_2}{\boldsymbol{S}^{\mathrm{T}}}} \end{array}} \right]$$

According to the basic Kalman filter theory, the least-squares estimate in (25) can also be computed by the standard Kalman filter algorithm in (2), but replacing ***W***_*k*−1_ and ***V***_*k*_ with ***QM***_1_***Q***^T^ and ***SM***_2_***S***^T^, respectively; this is exactly the formulas in (15) with (14). Insert the estimate in (25) back into (23), then back into (22), one can get the estimate of the normalized noises; this is exactly the formulas in (11). Insert the estimates of these noises into (21), one can update the two variance-component matrices; this is exactly the formulas in (13). So, the proposed method presented in Sect. 2, as the solution to the problem of minimizing (20), is proved.

Due to the nature of nonlinearity of the models, the first attempted estimate in (25) may not be the exact minimizer of (20); so, iterations may be needed. This is often called the IRLS iterations, see e.g^29^. Iterative algorithms other than the IRLS can also be employed; the Newton-Raphson algorithm is an example^19^. However, as shown in this contribution, in each iteration with the IRLS, the formulas are of the same forms with the standard Kalman filter.
